# Analysis of Epithelial-Mesenchymal Transition Metabolism Identifies Possible Cancer Biomarkers Useful in Diverse Genetic Backgrounds

**DOI:** 10.3389/fonc.2020.01309

**Published:** 2020-07-30

**Authors:** Meztli Matadamas-Guzman, Cecilia Zazueta, Emilio Rojas, Osbaldo Resendis-Antonio

**Affiliations:** ^1^Programa de Doctorado en Ciencias Biomédicas, UNAM, Mexico City, Mexico; ^2^Human Systems Biology Lab, National Institute of Genomic Medicine, Mexico City, Mexico; ^3^Departamento de Biomedicina Cardiovascular, Instituto Nacional de Cardiología-Ignacio Chávez, Mexico City, Mexico; ^4^Department of Genomic Medicine and Environmental Toxicology, Institute of Biomedical Research, UNAM, Mexico City, Mexico; ^5^Coordinación de la Investigación Científica—Red de Apoyo a la Investigación, UNAM, Mexico City, Mexico

**Keywords:** metabolism, EMT, glutathione, mathematical model, biomarker

## Abstract

Epithelial-to-mesenchymal transition (EMT) relates to many molecular and cellular alterations that occur when epithelial cells undergo a switch in differentiation generating mesenchymal-like cells with newly acquired migratory and invasive properties. In cancer cells, EMT leads to drug resistance and metastasis. Moreover, differences in genetic backgrounds, even between patients with the same type of cancer, also determine resistance to some treatments. Metabolic rewiring is essential to induce EMT, hence it is important to identify key metabolic elements for this process, which can be later used to treat cancer cells with different genetic backgrounds. Here we used a mathematical modeling approach to determine which are the metabolic reactions altered after induction of EMT, based on metabolomic and transcriptional data of three non-small cell lung cancer (NSCLC) cell lines. The model suggested that the most affected pathways were the Krebs cycle, amino acid metabolism, and glutathione metabolism. However, glutathione metabolism had many alterations either on the metabolic reactions or at the transcriptional level in the three cell lines. We identified Glutamate-cysteine ligase (GCL), a key enzyme of glutathione synthesis, as an important common feature that is dysregulated after EMT. Analyzing survival data of men with lung cancer, we observed that patients with mutations in GCL catalytic subunit (GCLC) or Glutathione peroxidase 1 (GPX1) genes survived less time than people without mutations on these genes. Besides, patients with low expression of ANPEP, GPX3 and GLS genes also survived less time than those with high expression. Hence, we propose that glutathione metabolism and glutathione itself could be good targets to delay or potentially prevent EMT induction in NSCLC cell lines.

## Introduction

Epithelial-mesenchymal transition (EMT) is a natural cellular phenomenon that converts epithelial cells to mesenchymal-like, giving them motile, and invasive properties (1). This process induces changes at different cellular levels, such as morphology (loss of apical-basal polarity), surface markers (cadherins), cytoskeleton (production of vimentin), transcriptional factors (Twist, Snail), miRNAs (miR-200), and metabolic pathways, among others (2, 3). EMT is a natural phenomenon that occurs in embryogenesis, wound healing, and some diseases like fibrosis and cancer. In cancer, EMT contributes to tumor cell survival, migration, invasion, and therapy resistance. Although EMT is a general phenomenon among many cancers, the cellular context and genetic background determine how this process is carried out (4). Furthermore, metabolic rewiring is essential for the induction of EMT (1, 5, 6). However, only specific enzymes and metabolites have been recognized as necessary for EMT induction (3, 5, 6), and most of them in specific genetic backgrounds and conditions.

Constraint-based modeling integrates a set of algorithms that combine computational modeling of genome-scale metabolic reconstruction with omic data to differentiate and predict metabolic alterations in biological systems (5). Among these computational tools, flux balance analysis (FBA) has been proven to be a powerful approach to explore and design metabolic phenotypes that range from the human microbiome to tissue-specific cancer cells (7, 8). Despite this approach has served as a guide to understand and design metabolic phenotypes, FBA does not correlate metabolites concentrations with metabolic fluxes. More specifically, under the steady-state situation, the null space of the stoichiometric matrix supplies with the available space of metabolic fluxes, leaving outside the metabolome profiles associated with a physiological state. To overcome this limitation, there have been suggested a variety of formalisms with capacities to move from the steady-state condition and explore the association between the concentration of metabolites and the phenotype state of the network. Among these formalisms, Dycone is a useful tool to infer relative changes in the enzyme regulation between two physiological conditions. It begins with a metabolic reconstruction and the concentrations of the metabolites in two conditions (9, 10). Unlike other approaches, Dycone does not assume specific kinetic parameters but calculates the space of all feasible kinetic constants (called k-cone) that mathematically ensure the measured concentrations of metabolites. Thus, the comparison between two kinetic spaces allows us to identify metabolic reactions whose activity potentially differentiates between conditions. Subsequently, the results could be connected to other omic data, such as genome or proteome.

In this paper, we analyzed the metabolic alterations in metabolism that follow EMT induction. Our study used Dycone to predict alterations in enzyme activity during EMT in three non-small cell lung cancer (NSCLC) cell lines (A549, HCC827, NCI-H358), using available metabolome data (11). These cell lines have different genetic backgrounds, hence we aimed to identify the metabolic similarities among them. Glutathione metabolism was one of the most affected pathways in all of the cell lines, according to the model. Furthermore, using microarray data we identified an alteration in the expression of genes involved in glutathione metabolism. These results suggested that glutathione metabolism plays an important role during EMT, thus we looked for survival data of lung cancer patients to observe the significance of these genes. Survival data from male patients with lung cancer showed that mutations in GCLC, and GPX2, key genes in glutathione metabolism, contribute to a bad prognosis. Additionally, the high expression of GPX2 and the low expression of ANPEP, and GPX3 also reduced the survival time of men with lung cancer. Based on these findings, we proposed GCLC, glutathione peroxidases, and glutathione as possible targets to regulate EMT in non-small cell lung cancer.

## Materials and Methods

Microarray and metabolome data was obtained from Sun et al. (11). All code used for analysis is available upon request. All data was significant when *p*-value was lower than 0.05 and log fold change >1, unless otherwise indicated.

### Microarray and Metabolome Data Analysis

Metabolome and microarray data was analyzed with R 3.5.3. Metabolites concentrations were converted to log scale and compared using a *t*-test.

Microarray data was normalized using the R package fRMA from Bioconductor and analyzed with the R limma package, unless otherwise indicated data was considered significant with a p-value lower than 0.05 and absolute fold change higher than 1. Code is available upon request.

### Enrichment Analysis

Enrichment analysis for metabolome data was done in MSEA web platform with the concentration table of each of the cell lines (A549, HCC827 and NCI-H358) (12, 13). The data was normalized by the median, log transformed, and scaled by mean-centered algorithm. We used “Enrichment analysis” to find enriched pathways, using Pathway-associated metabolite sets (SMPDB). We considered only the pathways with Holm P value below 0.05 and FDR below 0.05. We intersected the results of the three cell lines and identified those that were enriched in the three pathways.

### Network Reconstruction

To investigate the metabolic reprogramming before and after EMT in a system-level approach, we have constructed a medium-size but essential metabolic network (Supplementary Figure 2), which includes 74 metabolites and 112 reactions that represent our cell lines. The metabolic reactions were selected according to the following criteria:

1. Reactions had to be included in enriched metabolic pathways.
2. Reactions which had concentrations measured for substrates, products, or both.
3. Reactions that connected the network and are not isolated from others.
4. Reactions important to complete gaps even though substrates or products are not in data.
5. Finally, adjacent reactions where intermediates did not have measured concentrations were joined in a single reaction.

### Computational Modeling of *k-cone* Space

In order to identify the set of reactions that potentially change their metabolic activity in EMT, we analyzed metabolic data as in Diener et al. (10), using the R package Dycone (https://github.com/cdiener/dycone) (9, 10). Briefly, the algorithm assumes that all the metabolic reactions in reconstruction obey the law-mass action, and the system has reached a steady-state condition. Under these constraints, the feasible space of metabolic phenotypes before and after the EMT can be obtained through the null-space if the equation

(1) $$\begin{array}{r} S\;\cdot \;M\;\cdot \;k\;=\;0 \end{array}$$

where S indicates the stoichiometric matrix and *M* is a diagonal matrix whose entries are defined by

(2) $$\begin{array}{r} M_{i}\left(x\right)\;=\;\underset{j}{\prod }x_{j}^{S_{ji}} \end{array}$$

Here, *x*_*j*_ denotes the *j-esime* metabolite concentrations as *k* represents a vector containing all the kinetic parameter associated with each metabolic reaction. Taking into account the set of metabolites obtained before and after the EMT, we substitute these metabolome profiles separately on equation 2 for then obtain the null-space of equation 1 by independently. After this step, differential metabolic activity in EMT was obtained by comparing the null-spaces associated with before and after the EMT as described in (10). This analysis was performed with R version 3.5.3 as in the original paper with dycone package. All code used for analysis is available at https://github.com/Meztlimg/EMTanalysis.

### Survival Analysis

Genomic Data Commons Data Portal was used for the survival analysis of patients with mutations (https://portal.gdc.cancer.gov/). We used the portal to search survival data from male patients with bronchus and lung cancer as a primary site. We compared two cohorts of patients, the first patients with mutations in genes of interest and the second patients without mutations on these genes. Cumulative survival time was calculated by the Kaplan–Meier method and analyzed by log-rank testing. Survival analysis of patients with different expression levels was obtained from Kaplan Meier plotter (14). We used only male lung cancer patients. Cumulative survival time was calculated by the Kaplan–Meier method and analyzed by Cox univariate analysis. All analyses were performed at a significance level of 95% (*p* < 0.05).

## Results

### Different NSCLC Cell Lines Have Similarities in Metabolic Rewiring After EMT

**Figure 1:**
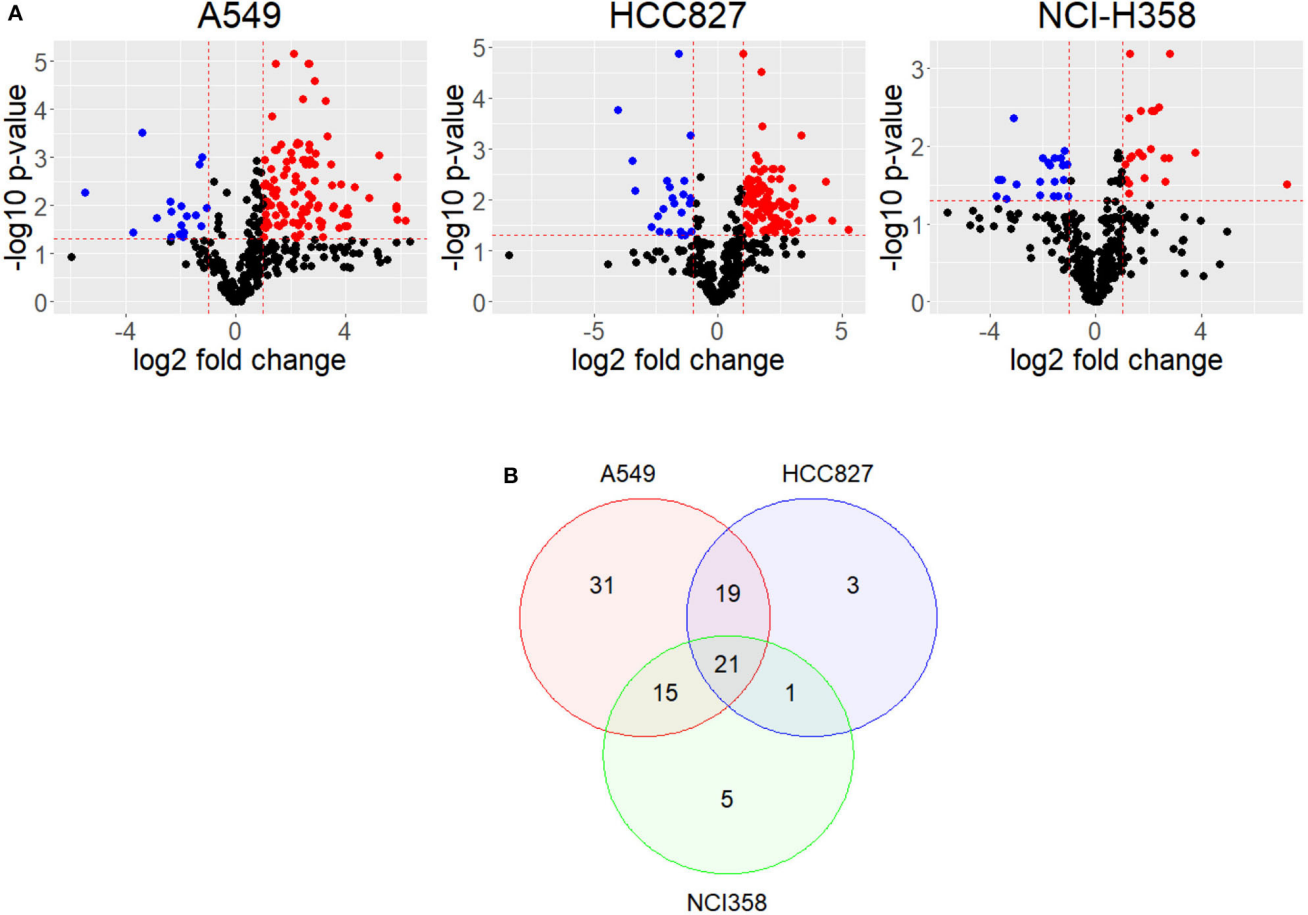
Metabolites have different concentrations after EMT. **(A)** Volcano plot shows differential concentrations of metabolites after EMT for each cell line. Red dots are more concentrated after EMT, while Blue dots are less concentrated. **(B)** Venn Diagram of enriched pathways in common between cell lines.

Upon EMT activation, cellular metabolism rewires to fulfill the new requirements of the cell (15–17). Alterations in the metabolome profile reflect how metabolism changes after a cellular process; therefore, we compared metabolomic public data of cell lines before and after EMT induction (11). This data comprises metabolome profiles from three different non-small cell lung cancer (NSCLC) cell lines (A549, HCC827, and NCI-H358) with different genetic backgrounds. A549 and NCI-H358 have mutations in KRAS, HCC827 has mutations in EGFR. To find key EMT features, we compared the three cell lines data and found their similarities. First, we calculated the log-fold change and identified the metabolites that significantly changed their concentration before and after EMT induction, for each cell line (Figure 1A). The metabolic profile associated with EMT does not present common patterns between cell lines. The analysis showed only six metabolites altered after EMT among the cell lines (Supplementary Figure 1). Despite EMT being experimentally triggered with TGF-β in all the cell lines, we found a lot of differences in metabolic concentration profiles. Presumably, these differences can emerge as a consequence of the variations in the genetic background of each cell line. Hence, we looked for a core of pathways that changed the three NSCLC cell lines. We performed an enrichment analysis of each of the cell lines before and after EMT using metabolomic data, and then compared the results. In summary, our study allowed us to conclude that 21 pathways had similar differences in all three cell lines after induction of EMT (Figure 1B and Supplementary Tables 1–3). We identified some pathways related to amino acids metabolism, such as alanine, glycine, serine, and glutamate, among others, according to other studies (11, 15, 17, 18). Furthermore, we discovered that additional pathways such as Fatty Acid metabolism, Citric Acid Cycle, and glutathione metabolism also changed after EMT (Supplementary Table 13). We noticed that A549 is the cell line with the highest number of altered pathways characterizing the EMT respect to other cell lines, and simultaneously it contains most of the metabolic pathways enriched in NCI-H358 and HCC827 (Figure 1B). We concluded that EMT is accompanied by two types of metabolic rewiring, the first defined by a core of metabolic transformations common for different cell lines, and others characterizing cell type and its particular genetic context.

### Metabolic Reconstruction Identified Specific Reactions Altered After EMT

**Figure 2:**
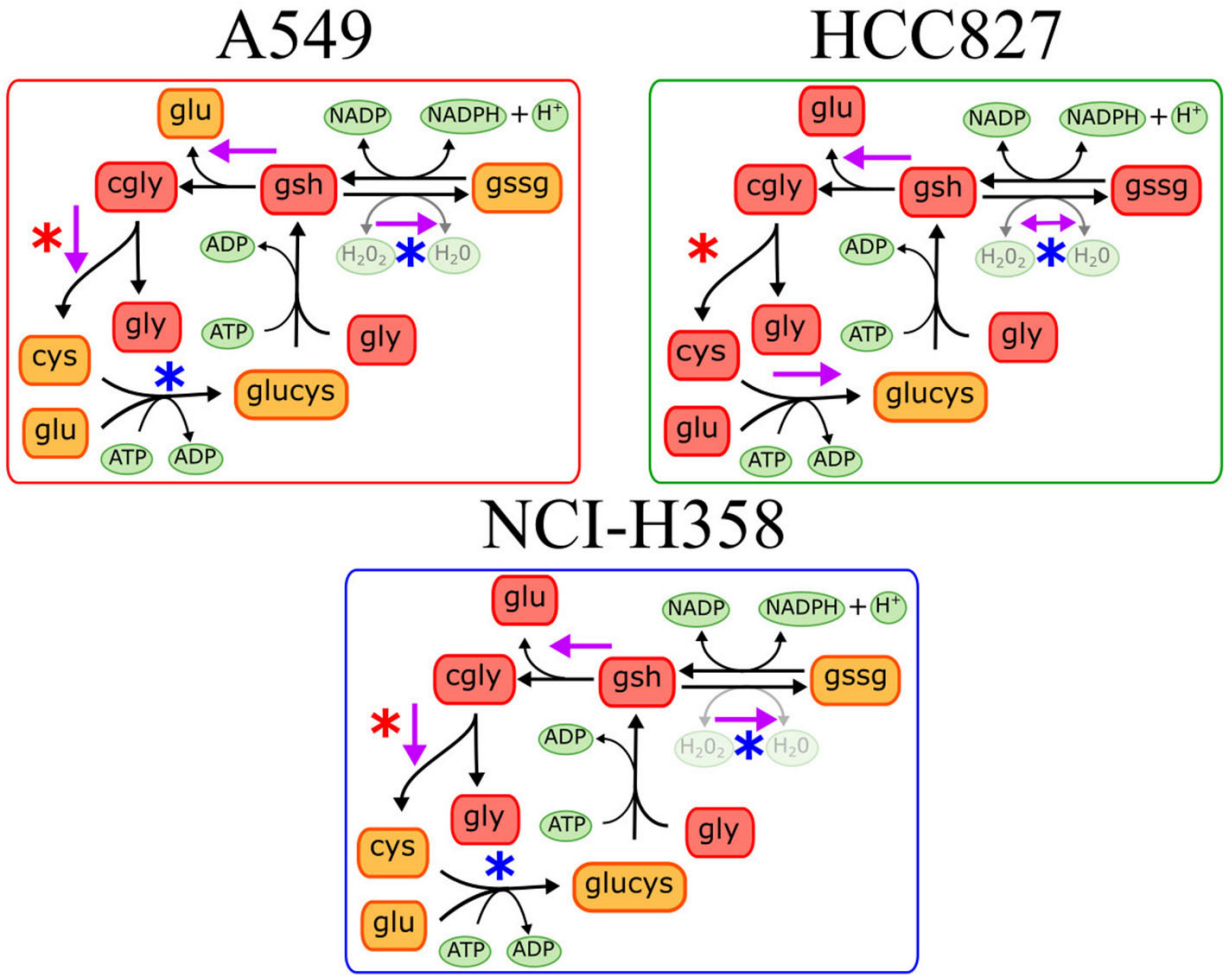
Predictions on glutathione metabolism in the three different cell lines. Purple arrows indicate the predicted metabolic reactions with a significant enzymatic change during EMT. Blue asterisks indicate downregulation of genes coding the corresponding enzyme, while red asterisks indicate gene overexpression after EMT induction.

We found different metabolic pathways enriched with alterations on metabolite concentrations, however, it is difficult to locate which reactions or mechanisms are underlying the metabolic rewiring in EMT. Identifying these enzymatic alterations and control reactions during EMT is a valuable task given their implication in targeted drug design against metastasis. To this end, constraint-based modeling has shown to be a proper paradigm in systems biology to find metabolic targets in cancer (5, 9, 19). In order to identify the critical reactions changing the concentrations of metabolites during EMT, we reconstructed a network to represent the main reactions affected in the three cell lines, based on the enrichment analysis. During the reconstruction, we only consider those reactions that had metabolites measured in the metabolomic data, and those which were connected to the rest of the network (Materials and Methods). Overall, our reconstruction had 112 reactions and 74 metabolites that included glycolysis, TCA cycle, amino acid metabolism, pentose phosphate, and glutathione metabolism (Supplementary Figure 2). The absence of some metabolites concentrations impeded the addition of the rest of the enriched pathways. Mathematical representation of this reconstruction was our cornerstone to explore the metabolic capacities that support the phenotype underlying EMT, through a myriad of computational methods included in constraint-based modeling (8, 20). Among this set of available methods, Dycone is a computational approach that allowed us to identify enzymatic alterations in metabolic networks between two physiological conditions starting from their respective metabolomic profiles (10). Briefly, combining a metabolic reconstruction with the profiles of metabolites in two different conditions, Dycone allowed us to identify and postulate the changes on the metabolic activity before EMT and after EMT (Supplementary Figure 3). The model predicted that diverse reactions changed after EMT in each cell line, however, we focused on those sets of reactions that changed in the three NSCLC cell lines (Supplementary Tables 6–8). For example, the model predicted that glutaminase (GLS) reaction, which catalyzes the conversion of the amino acid glutamine to glutamate, changed in all the cell lines. In agreement, other studies have reported this enzyme as an essential metabolic feature of EMT in breast and lung cancer (11, 21). Furthermore, our model also predicted the alteration of glutathione metabolism in all of the three cell lines. Specifically, glutathione oxidation, reduction, and degradation changed in all the cell lines, whereas glutathione synthesis only in HCC827 (Figure 2). These results agree with other studies reporting glutathione peroxidases and other enzymes related to glutathione metabolism as critical players during EMT in different types of cancer, such as melanoma, cervical, hepatocellular, gastric and pancreatic cancer (18, 22–26).

### EMT Alters the Expression of Metabolic Genes in the Three Cell Lines

Integrating transcriptional data with our model may lead us to a better understanding of the metabolic mechanisms involved in EMT. To get an insight into the role of glutaminase and glutathione metabolism in the EMT, we analyzed the expression of genes that code enzymes involved in these reactions. This data came from the same study as the metabolomic data, letting us relate easily the model predictions with the expression analysis. Glutaminase, the rate-limiting enzyme of glutamine to glutamate, is coded by GLS and GLS2 genes in humans. GLS was upregulated after EMT in A549 and NCI-H358, while GLS2 was downregulated after EMT in HCC827 (Supplementary Figure 4). These differences could be because the cell lines have different genetic backgrounds that could affect the basal expression and regulation of these genes. As we know, each genetic background determines how each EMT-TF promotes distinct metabolic alterations (18). Nonetheless, EMT suppresses the GLS2 gene in breast cancer, promoting glutamine independence, less mitochondrial activity, high metastasis, and low survival to patients (21). This could also explain the accumulation of glutamine in all the cell lines after EMT. However, the expression of GLS is necessary for the induction of EMT in breast and colorectal cell lines (27, 28). Hence, both changes in expression in the different cell lines are logical, as we were observing the effects after the EMT, and we could not decipher the order of the events with this data. Besides, NSCLC cell lines have been reported as sensitive to GLS inhibitors after EMT, confirming the importance of this reaction in this phenomenon (29). In conclusion, as either our model and gene expression analysis matched identifying an alteration in this reaction, we propose that the regulation of glutaminase reaction is highly important in EMT.

**Figure 3:**
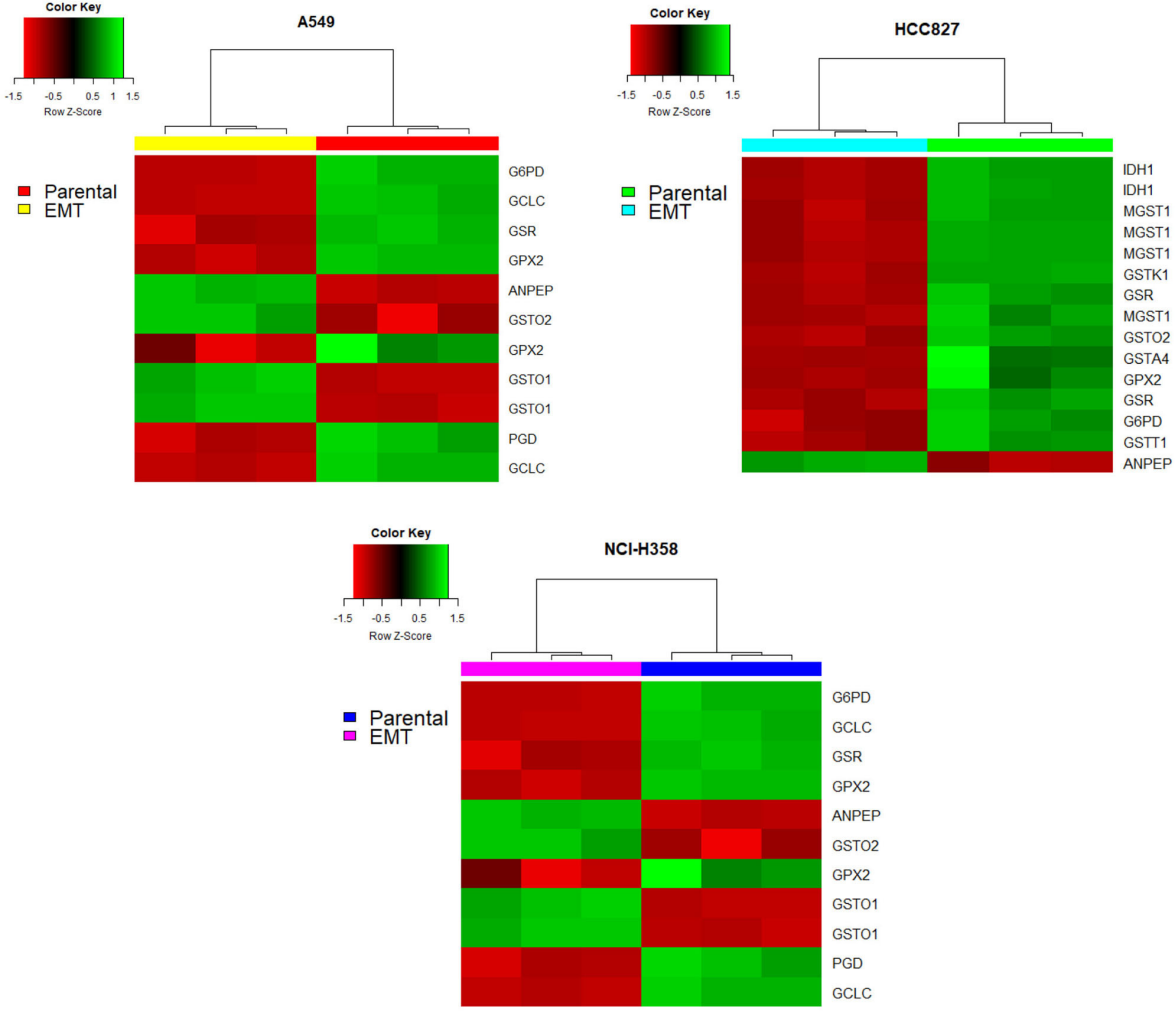
Glutathione pathway genes had different expressions after EMT in all the cell lines. Red indicates a downregulation while green indicates an overexpression. In all heatmaps the right part corresponds to before EMT induction (parental cell lines) and the left part corresponds to after EMT induction.

On the other hand, glutathione metabolism has three stages, synthesis, degradation, and glutathione oxidation/reduction to process ROS. Our model predicted that this pathway changed in different ways among the three NSCLC cell lines. The predictions indicated that EMT affects glutathione degradation in all of the cell lines, while glutathione synthesis changed only in A549. Consequently, we analyzed the expression of genes belonging to this pathway in the three NSCLC cell lines. We found a downregulation of Glutathione peroxidase 2 (GPX2) and Glutathione reductase (GSR), plus an upregulation of Alanyl Aminopeptidase (ANPEP) after EMT in the three cell lines (Figure 3). GPX and GSR detoxify the cells using glutathione to metabolize hydrogen peroxide, whereas, ANPEP catalyzes the last step of degradation of glutathione. Several studies have described that GPX genes are critical for EMT, either preventing or enhancing it (23–25). Specifically, GPX2 downregulation is contrasting because it has been reported as a regulator of EMT markers, enhancing invasion, and metastasis targeting the Wnt pathway in cervical and pancreatic cancer (26, 30). Nevertheless, NSCLC cells usually have an aberrant Wnt pathway, explaining why this could be different in these cells (31). Moreover, glutathione reduction has also been reported as a key player in the induction of EMT lowering oxidative stress and enhancing metastasis in murine melanoma cells (32), although our cells showed a downregulation of GSR gene. This could be specific for murine animals, or because in our case, TGF-β induced EMT and elicits other specific mechanisms to regulate oxidative stress. Moreover, glutathione biosynthesis is also important on glutathione metabolism, and it has been described as a critical process during EMT induction in different epithelial cell lines (33–36). Glutamate-cysteine ligase (GCL) is the first rate-limiting enzyme of glutathione synthesis. GCL has two subunits, the catalytic and the modifier subunits, coded by GCLC and GCLM, respectively. Based on the metabolomic data, the model predicted that EMT altered GCL reaction on HCC827, however, the transcriptional data showed downregulation of GCLC on A549 and NCI-H358. This result and previous studies in other epithelial cell lines suggest that GCL needs to be reduced to lower glutathione levels for EMT induction, playing an important role in metabolic rewiring (22, 33, 34, 37). In conclusion, integrating the model predictions and expression data gave us enough information to find useful targets important for EMT development on NSCLC cells, suggesting downregulation of glutathione metabolism, specifically GPX, GSR, and GCL, which could be key features in the metabolic rewiring during EMT.

### Mutations on Glutathione Metabolism Genes Affect Prognosis

**Figure 4:**
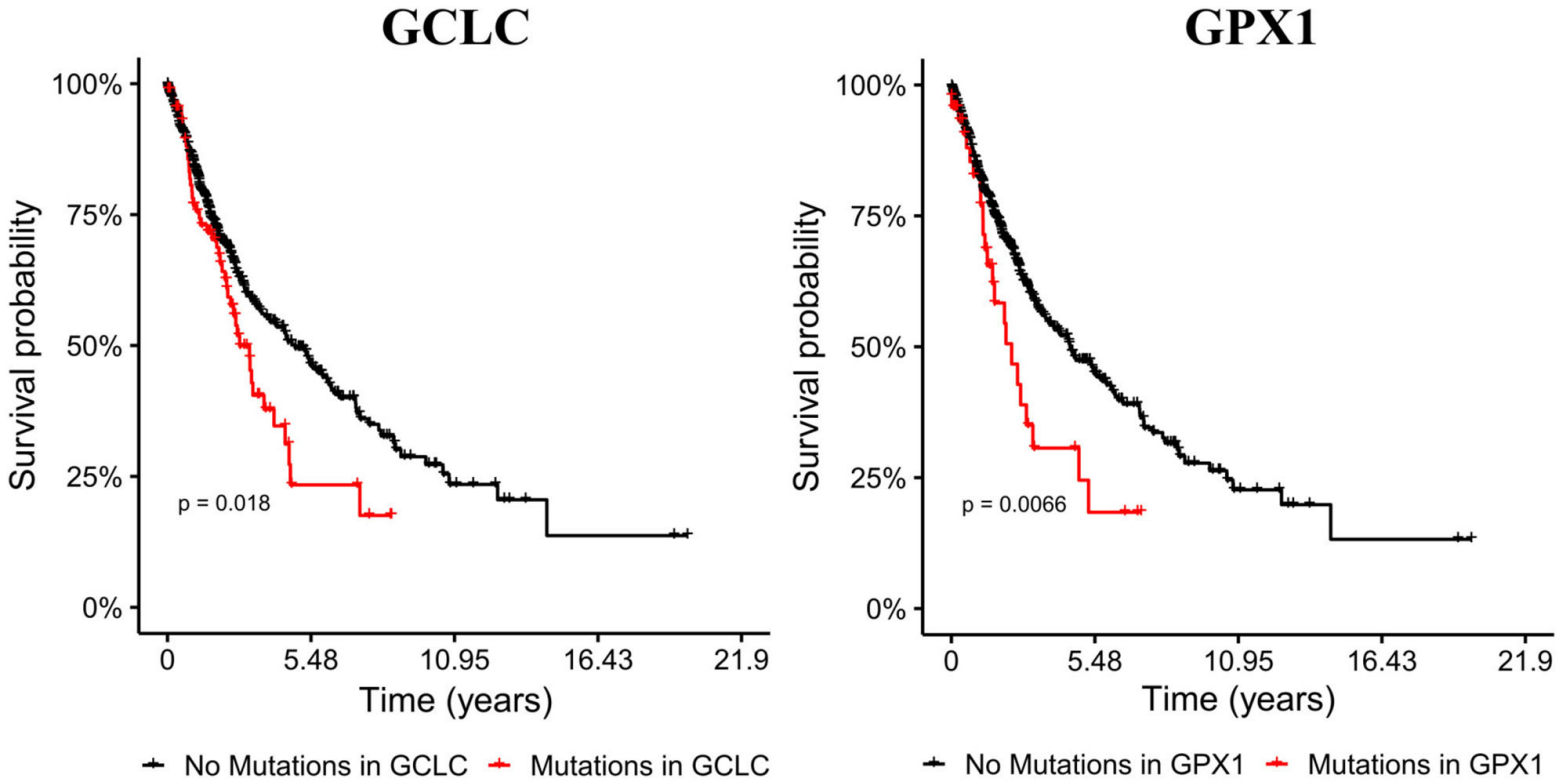
Mutations on GCLC and GPX1 genes reduce survival time of male patients with lung cancer. Cohorts with mutations on GCLC and GPX1 are shorter than the other cohorts. Left panel shows Kaplan-Meier survival curves of patients with (red) and without (black) mutations on the GCLC gene. Right panel shows Kaplan-Meier survival curves with (red) and without (black) GPX1 mutated.

EMT has been related to high metastasis and poor prognosis, thus we evaluated the impact of mutations in metabolic genes in the survival of male patients with bronchus and lung cancer. Our target genes were primarily GLS, GSR, GCLC, and GPX genes, which our model and transcriptional data predicted as key players in EMT induction for all the NSCLC cell lines. We selected cohorts of patients with bronchus and lung cancer from the TCGA database (38), and compared the survival time to see if mutations on genes affect prognosis. This analysis showed that male bronchus and lung cancer patients with mutations on the GCLC gene survived less time than those without this gene mutated (*p*-value 0.0183) (Figure 4). Besides, patients with mutations on GPX2 showed a little less survival but were not significant. Nevertheless, humans have seven GPX genes coded in their DNA, from all of them, we noted that only patients with mutations on the GPX1 gene showed less survival rate than those without mutations on this gene (Figure 4). This suggested that GCLC and GPX1 genes may be used as biomarkers of bad prognosis in people with lung cancer, even with different genetic backgrounds. Patients with mutations either on GLS or GSR had similar survival time compared with patients without these mutations (data not shown).

**Figure 5:**
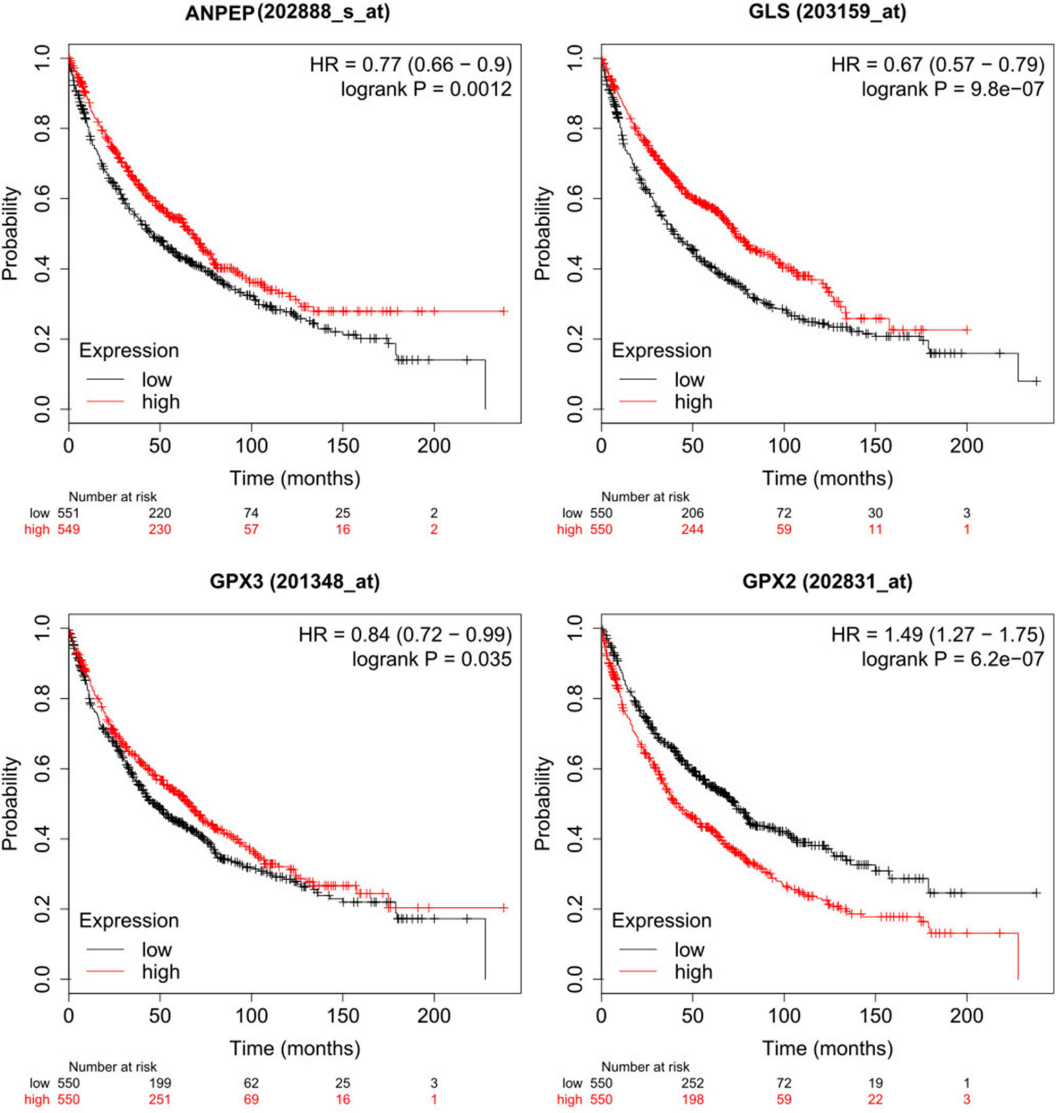
Low expression of ANPEP, GLS and GPX3 and high expression of GPX2 reduced survival on male lung cancer patients. High expression cohort in red and low expression cohort in black.

However, sometimes patients lack mutations on these genes, but transcription levels are altered, leading to a differential metabolism on these patients. Hence, we also analyzed survival data of patients with differential expression of genes of interest (14). In summary, patients with high expression of GPX2 survive less time than those with low expression of these genes (*p* = 6.2e−07), while patients with low expression of ANPEP, GPX3 and GLS survive less time than those with high expression (*p* = 0.0012, 0.035, 9.8e−07, respectively) (Figure 5). Overall, these findings suggested that glutathione metabolism is a key player in cancer prognosis and could be used as a biomarker for poor survival in men with lung cancer. More studies need to be done to understand the role of glutathione metabolism genes and the associated mechanisms in cancer and EMT. As a result of these observations, we postulate that GCLC, GPX1 genes and glutathione metabolism are possible targets to delay EMT or potentially prevent the EMT in lung cancer. This last hypothesis will be a future perspective of this work.

## Discussion

Epithelial to mesenchymal transition (EMT) in cancer is one of the first steps for metastasis and drug resistance. EMT induction rewires metabolism, and some metabolites and enzymes have been identified as essential features for this process, such as the accumulation of fumarate, changes of succinate dehydrogenase and high levels of ROS in the cell, among others (3, 5, 6, 11, 16). However, differences in genetic background promote distinct metabolic alterations; hence it is essential to find common metabolic alterations between cancer cell lines that can be used as possible biomarkers of therapeutic targets (5, 18). This paper fundamentally used the metabolome and transcriptome data previously reported for the EMT triggered by TGF-β in three NSCLC (11). Consequently, this experimental condition shaped most of our conclusions. As a central contribution of this paper, we show how these paired technologies supplied us with a valuable scheme to calibrate and assess the predictions coming from our computational model. Although TGF-β is not the only signal to induce EMT, our computational framework and analysis is a valuable scheme to understand the underlying metabolic mechanisms of EMT induction, generate testable hypotheses, and design strategies to study the process under another stimulus. Despite a metabolic signature that characterizes EMT in different cell lines has been described (18), metabolite concentrations before and after the transition showed very different profiles for each of the cell lines analyzed. Based on a metabolic enrichment analysis, we conclude that metabolic alterations during EMT can be split into two classifications. First, a core of metabolic pathways commonly altered in the three cell lines. Second, a set of metabolic pathways whose enrichment is cell-type-specific whose metabolic profile depends on factors such as the genetic background of the cell. For example, KRAS is known to affect metabolism when mutated on different types of cancers (39–41). Overall, our metabolic enrichment analysis indicated specific pathways altered in all the three NSCLC cell lines (Supplementary Tables 1–3). Among those pathways, we found Glycolysis, Krebs cycle, Amino acids metabolism, and glutathione metabolism. These results agree with other studies reporting alterations on these pathways on breast cancer, among others (5, 21). To identify the enzymatic activity that accompanied the EMT, these pathways were used to accomplish a metabolic reconstruction to explain the differences in metabolites concentrations after EMT induction (9, 10). As a result, our *in silico* approach predicted that some set of reactions changed in all three cell lines. One of these reactions was glutaminase (GLS), which we expected as the original paper showed impairment in the conversion of glutamine to glutamate (11). Notably, we also observed an alteration on the expression of GLS in all of the cell lines. In agreement, GLS has already been reported as a critical enzyme for EMT induction in breast cancer and essential for migration properties in colorectal cancer (6, 11, 27).

On the other hand, the model predicted that reactions involved in glutathione metabolism changed in the three cell lines, paired with an alteration of the expression of genes encoding enzymes involved in this pathway. Glutathione oxidation and reduction are usually carried out by glutathione peroxidases (GPXs) and glutathione reductase (GSR). At the transcriptional level, EMT downregulated the expression of GPX2 and GSR in A549, HCC827, and NCI-H358 cell lines. Furthermore, GSR and GPX genes have already been reported as essential features to block EMT in melanoma cells, cervical cancer, hepatocellular carcinoma, gastric, pancreatic, breast, and colon cancers (23–26, 30, 42, 43). Hence, these findings supply evidence that the downregulation of GPX2 and GSR may be necessary for EMT induction on NSCLC cell lines. In concordance, survival analysis displayed that mutations, or low expression of GPX genes predict a poor prognosis for male lung cancer patients, showing the possible importance of these genes in cancer development, possibly making this disease more malignant.

Some studies report that glutathione and ROS levels are critical factors for EMT induction in lung cancer, lens epithelial cells, and TGF-β induced fibrogenesis (22, 34, 36, 44). Therefore, glutathione synthesis and degradation must be critical during EMT development. ANPEP, a gene responsible for the last steps of degradation of glutathione, showed downregulation in the three cell lines, a finding that corresponded with our *in silico* metabolic analysis of A549 and NCI-H358. ANPEP has been correlated with EMT markers at the proteome level, finding higher amounts of this protein when EMT markers are highly produced, however, this study is comparing two different cell lines with different genetic backgrounds, and they do not induce EMT in any form (45). Nevertheless, this indicates that somehow this protein participates in EMT. Also, ANPEP is part of the mesenchymal markers of Mouse mammary carcinoma cell line induced to EMT with TGF-β (46). In conclusion, we propose that ANPEP has an essential role during EMT in NSCLC cell lines, but more studies need to be done to understand the mechanisms involved.

Following, as the model indicated that glutathione synthesis was also a key feature of EMT, we investigated the behavior of Glutamyl-cysteine ligase (GCL). GCL catalyzes the first and rate-limiting step in the production of the cellular antioxidant glutathione. Our computational modeling predicted that GCL had changes in HCC827. Furthermore, GCLC, the gene that encodes the catalytic subunit of this enzyme, was downregulated in A549 and NCI-H358. From a clinical point of view, there is evidence that the high expression of GCLC predicts a better prognosis for melanoma patients (22). We also observed that patients of lung cancer with mutations on GCLC survive less time than those without mutations on this gene. Besides, GCLC has also been reported as a biomarker of poor survival in melanoma patients, and malignant phenotypes were more prominent in melanoma cells with lower GCLC expression (22). As well, GCLC has been found to overexpress in liver metastases of colorectal cancer and promotes cancer cell survival (47). Also, GCLC activation is associated with anti-tumor drug resistance in breast, lung, liver, head, and neck cancer (37, 48–50). Based on our *in silico* results and previous studies, we propose that both ANPEP and GCL have key roles in EMT development, however prognostic significance, as well as its potential as a pharmacological target, requires further in-depth investigations.

In conclusion, we presented a systems biology approach that integrates computational modeling, metabolome, and microarrays and can serve as a quantitative framework to predict and identify important reactions in EMT of NSCLC cell lines. These predictions are potentially useful in the design of therapeutic targets, as we compared NSCLC cell lines with different genetic backgrounds, hence having differences in their metabolism. Thus, finding similarities and differences between these cell lines help us understand the underlying mechanisms of the metabolic rewiring during EMT. Notably, the proposed genes depicted in this work have already been reported in other cancer as essential features for EMT induction. Overall, we highlight that our model can serve as a quantitative framework to predict and identify important reactions in EMT of NSCLC cell lines. Specifically, we highlight the role that glutathione metabolism has into EMT, a pathway that can be used to improve the outcome of patients in the future.

## Data Availability Statement

Publicly available datasets were analyzed in this study. This data can be found here: Sun et al., (11) Supplementary Material (https://cancerandmetabolism.biomedcentral.com/articles/10.1186/2049-3002-2-20). All code used for analysis is available at https://github.com/Meztlimg/EMTanalysis.

## Author Contributions

MM-G and OR-A developed the methods and MM-G performed the analysis. MM-G and OR-A wrote the paper. CZ and ER helped with the manuscript revision and writing. All authors contributed to the article and approved the submitted version.

## Conflict of Interest

The authors declare that the research was conducted in the absence of any commercial or financial relationships that could be construed as a potential conflict of interest.
