# Supplementary material for: Analysis of Epithelial-Mesenchymal Transition Metabolism Identifies Possible Cancer Biomarkers Useful in Diverse Genetic Backgrounds
Source: Front Oncol. 2020 Jul 30;10:1309. doi: 10.3389/fonc.2020.01309 (PMC7406688; doi:10.3389/fonc.2020.01309)
Supplement: Supplementary file 2 [file Data_Sheet_2.docx]

Supplementary Material

# Supplementary Data

## Supplementary Tables S1-S12

Supplementary Table S1 – MSEA enrichment results of A549

Supplementary Table S2 – MSEA enrichment results of HCC827

Supplementary Table S3 – MSEA enrichment results of NCI-H358

Supplementary Table S4 – Metabolome measurements for A549, HCC827 and NCI-H358 cells along with their KEGG compound and HMDB IDs.

Supplementary Table S5 – Reactions considered in the model for the central carbon metabolism.

Supplementary Table S6 – Dycone results for A549

Supplementary Table S7 – Dycone results for HCC827

Supplementary Table S8 – Dycone results for NCI-H358

Supplementary Table S9 – Sample IDs from the GEO data base and their associated cell type.

Mapping of the enzymes catalyzing the reactions in the model to ENTREZ gene IDs and their associated log fold changes and p-values from the dycone results and differential gene expression:

Supplementary Table S10 – A549

Supplementary Table S11 - HCC827

Supplementary Table S12 - NCI-H358

# Supplementary Figures and Tables

##
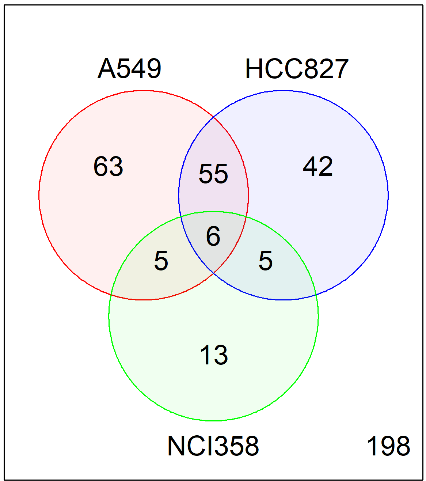
Supplementary Figures

| glycerol 3-phosphate (G3P) |
| --- |
| glycerophosphorylcholine (GPC) |
| Glycyltyrosine |
| N-acetylglucosamine 6-phosphate |
| nicotinamide ribonucleotide (NMN) |
| Tryptophan |

**Supplementary Figure 1.** Metabolites concentrations changed after induction of EMT. On the left a Venn diagram showing the number of metabolites with different concentrations after EMT induction. On the right, the table shows those metabolites that had differences in concentration for the three cell lines.


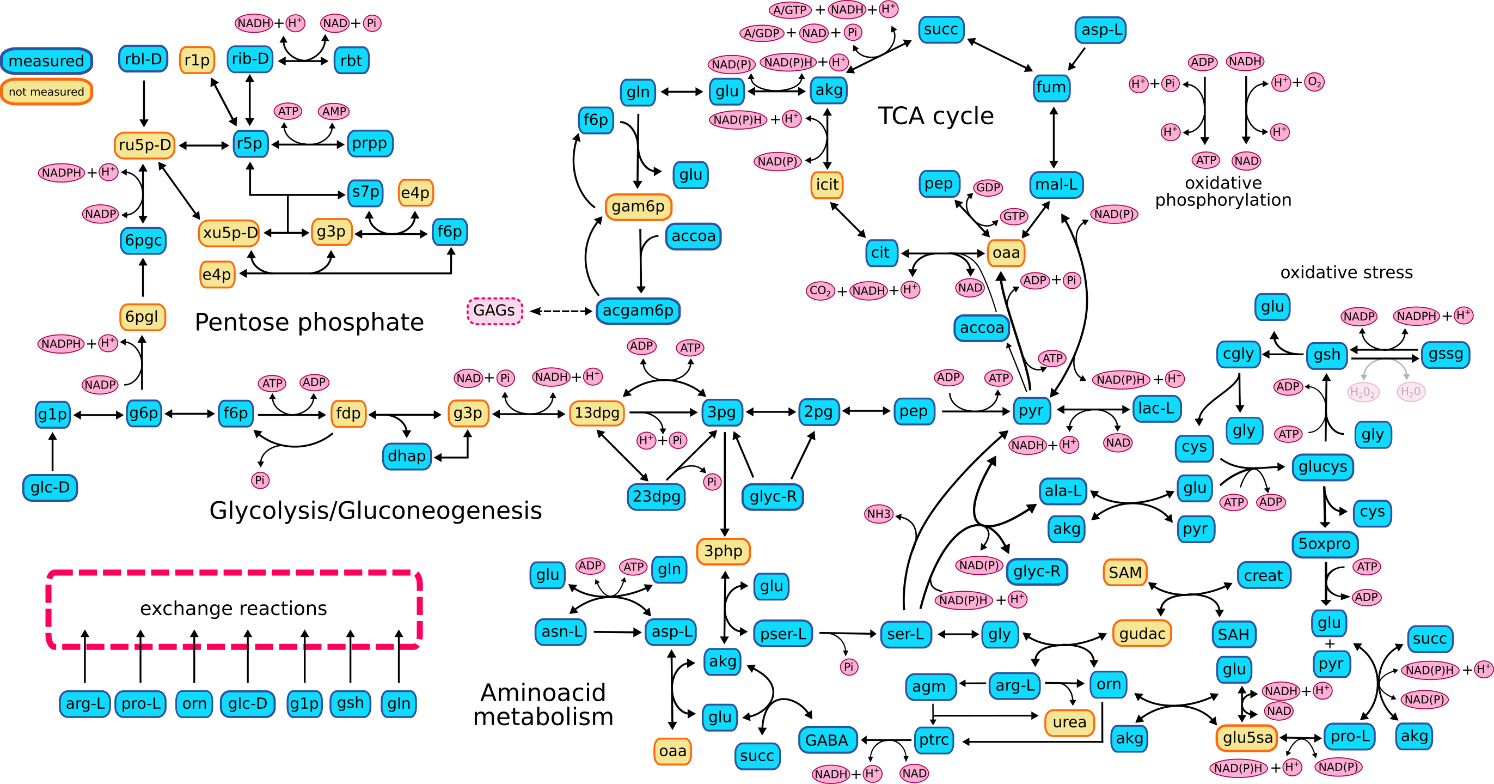


**Supplementary Figure 2.** Graphical representation of the metabolic reconstruction used for the analysis. It had 74 metabolites and 112 reactions. In blue metabolites with concentrations measured and in yellow those without concentrations registered on the metabolomic dataset.


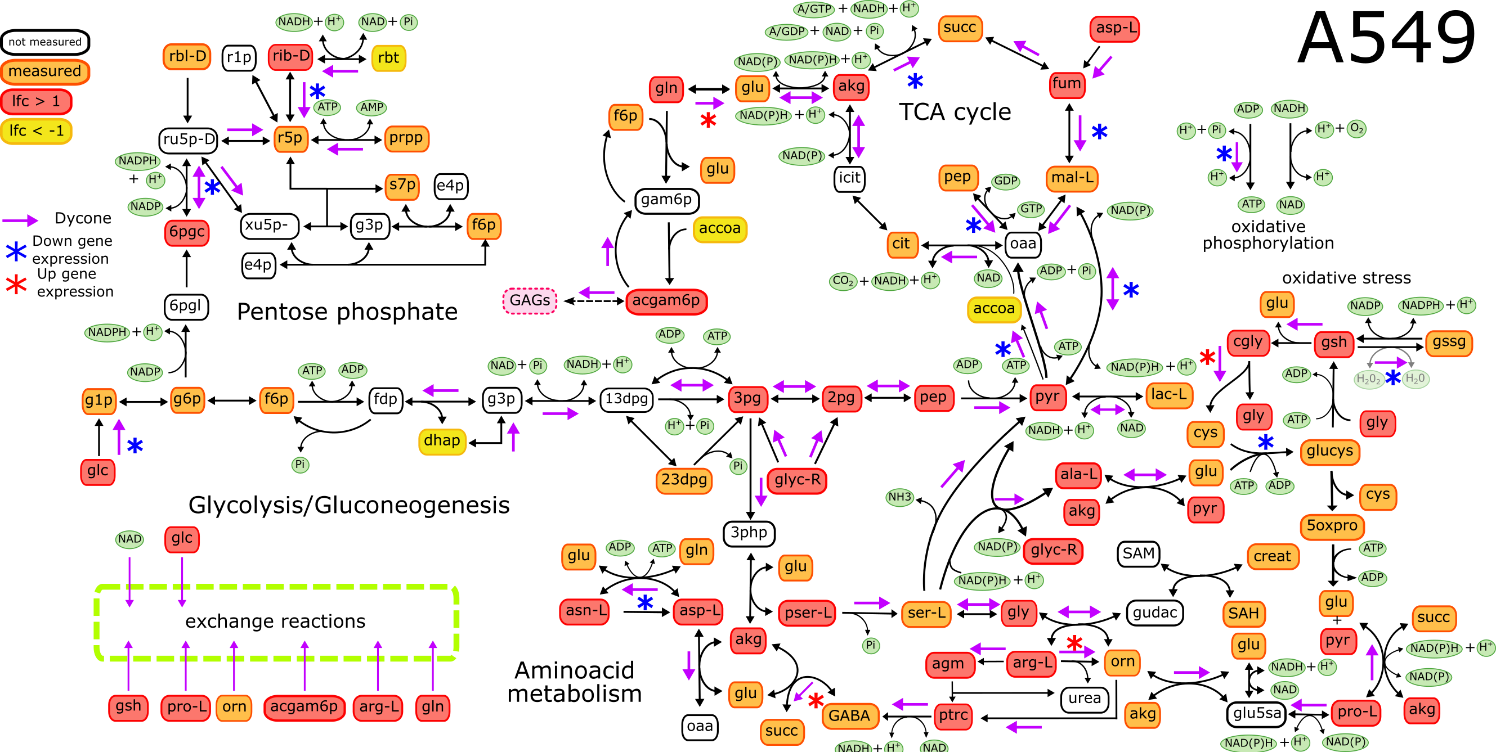


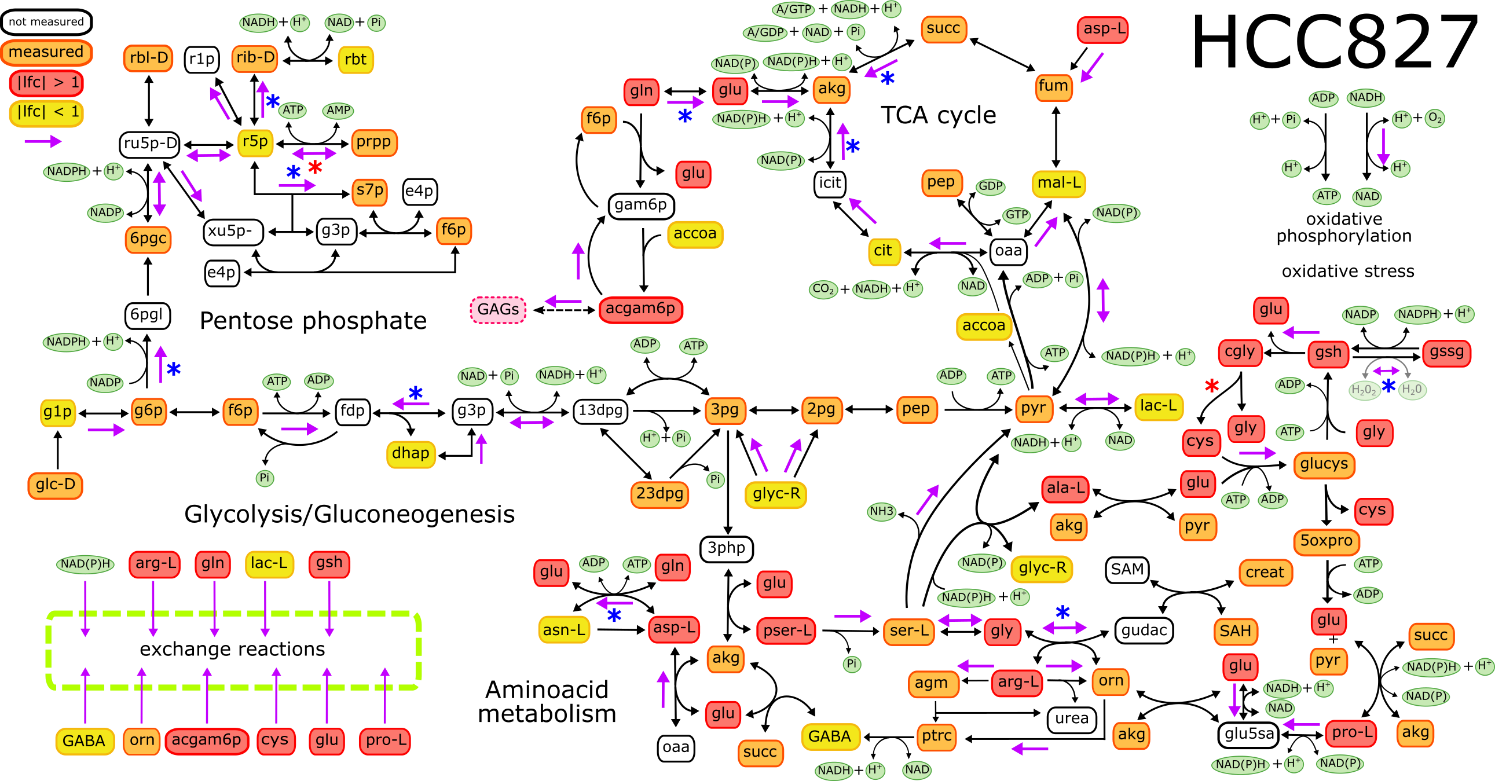


**Supplementary Figure 3.** Reactions alterations on A549 and HCC827. This graphical representation shows all dycone predictions (purple arrows) and differential gene expression (asterisks, red depicts upregulation, while blue depicts downregulation). Red metabolites had more concentration after EMT (|lfc| > 1), yellow metabolites had less concentration after EMT (|lfc| < 1), and orange maintained the same concentration. Lfc: log fold-change.


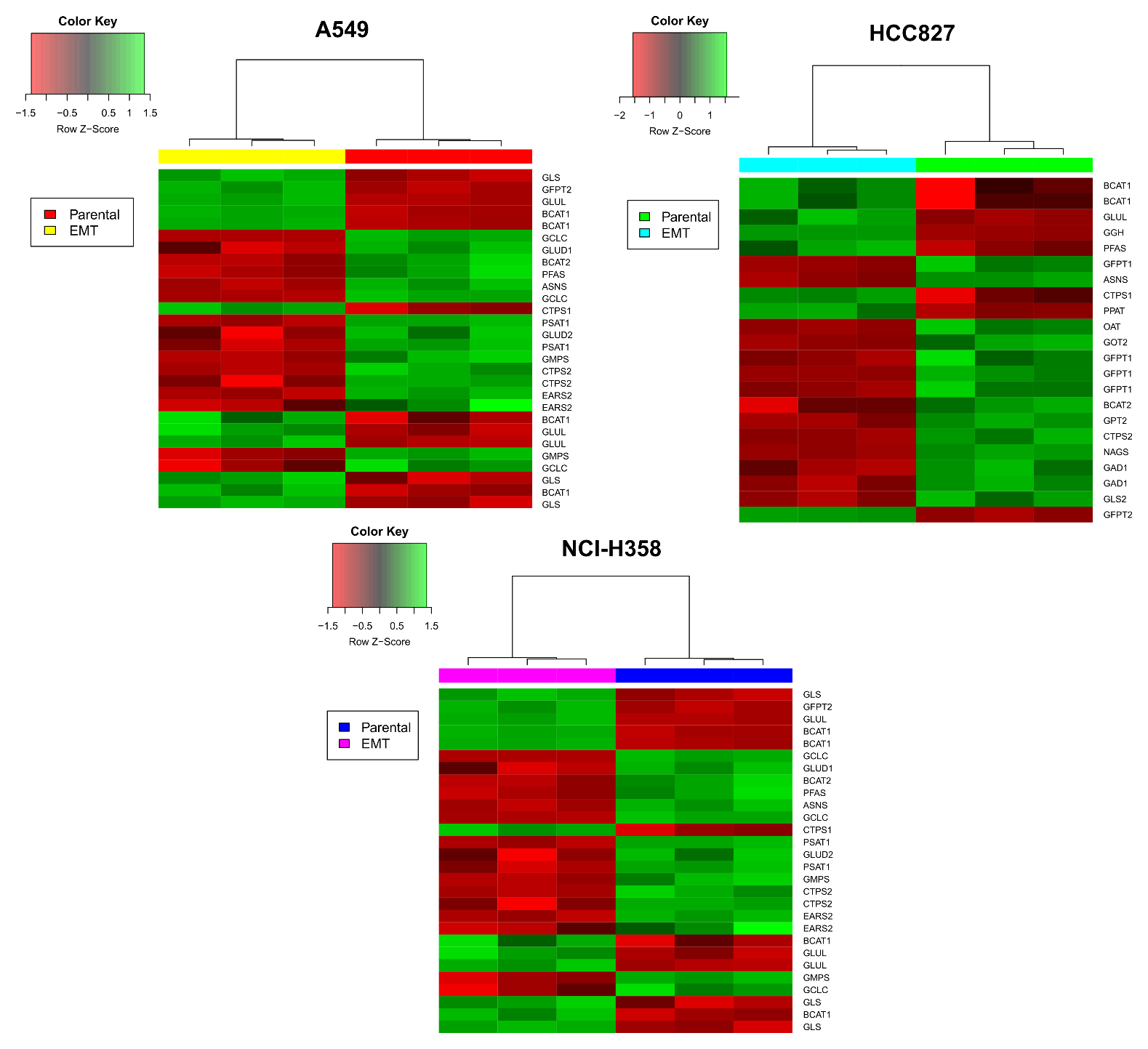


**Supplementary Figure 4.** Glutamate Metabolism genes had different expression profiles after EMT induction. Red indicates a downregulation while green indicates an overexpression. In all heatmaps the right part corresponds to before EMT induction (parental cell lines) and the left part corresponds to after EMT induction.

## Supplementary Table

**Supplementary Table 13. Pathways enriched with a *p* value less than 0.01 in the three cell lines**

Beta-Alanine Metabolism

Beta Oxidation of Very Long Chain Fatty Acids

Citric Acid Cycle

Ethanol Degradation

Fatty acid Metabolism

Folate Metabolism

Glutamate Metabolism

Glutathione Metabolism

Glycine and Serine Metabolism

Lysine Degradation

Methionine Metabolism

Mitochondrial Beta-Oxidation of Medium Chain Saturated Fatty Acids

Mitochondrial Beta-Oxidation of Short Chain Saturated Fatty Acids

Nicotinate and Nicotinamide Metabolism

Phytanic Acid Peroxisomal Oxidation

Purine Metabolism

Pyruvate Metabolism

Retinol Metabolism

Steroid Biosynthesis

Tryptophan Metabolism

Valine, Leucine and Isoleucine Degradation
